# PI3K/AKT is involved in mediating survival signals that rescue Ewing tumour cells from fibroblast growth factor 2-induced cell death

**DOI:** 10.1038/sj.bjc.6602384

**Published:** 2005-02-01

**Authors:** M Hotfilder, P Sondermann, A Senß, F van Valen, H Jürgens, J Vormoor

**Affiliations:** 1 Department of Pediatric Hematology/Oncology, University Children's Hospital, Münster, Germany; 2 Department of Orthopedics, 48129 Münster, Germany

**Keywords:** fibroblast growth factor 2, basic fibroblast growth factor, Ewing tumours, apoptosis, survival signals

## Abstract

While *in vitro* studies had shown that fibroblast growth factor 2 (FGF2) can induce cell death in Ewing tumours, it remained unclear how Ewing tumour cells survive *in vivo* within a FGF2-rich microenvironment. Serum- and integrin-mediated survival signals were, therefore, studied in adherent monolayer and anchorage-independent colony cell cultures. In a panel of Ewing tumour cell lines, either adhesion to collagen or exposure to serum alone only had a minor protective effect against FGF2. However, both combined led to complete resistance to 5 ng ml^−1^ FGF2 in three of four FGF2-sensitive cell lines (RD-ES, RM-82 and WE-68), and to an increased survival as compared to other culture conditions in TC-71 cells. Inhibition studies with LY294002 demonstrated that the serum signal is mediated via the phosphoinositide 3-OH kinase/AKT pathway. Thus, Ewing tumour cells escape FGF2-induced cell death by modulating FGF2 signalling. The tumour microenvironment provides the necessary survival signals by integrin-mediated adhesion and soluble serum factor(s). These survival signals warrant further investigation as a potential resistance mechanism to other apoptosis-inducing agents *in vivo*.

The molecular hallmark of the diverse group of Ewing tumours are chromosomal translocations leading to the fusion of the EWS gene on chromosome 22 with different members of the ETS family of transcription factors (de Alava and Gerald, 2000). The EWS/ETS fusion proteins appear (i) to act as abnormal transcription factors regulating a network of target genes (e.g., *manic fringe*, PDGF-C, TGF*β*II receptor) (Arvand and Denny, 2001) and (ii) to influence post-transcriptional gene processing by interacting with components of the cellular splicing machinery (e.g., TASR, SF1 and U1C) (Knoop and Baker, 2001). Both effects are thought to be important for the formation of the malignant phenotype. Despite the progress in unravelling the molecular biology of this tumour entity, the exact mechanisms that govern growth and survival of Ewing tumours *in vivo* are still poorly understood.

Fibroblast growth factor (FGF2) (formerly: basic fibroblast growth factor (bFGF)) is a classical growth and differentiation factor that is ubiquitously expressed and belongs to a family of heparin-binding, single-chain polypeptides. It is present in the extracellular matrix where it is bound to heparan sulphate proteoglycan. It influences a variety of biological processes including cell growth and differentiation and angiogenesis. High levels of FGF2 activity in tumour cells suggested a role in cell proliferation and tumour angiogenesis (Ornitz and Itoh, 2001). Introduction of an FGF2 cDNA expression vector in hamster kidney fibroblasts induced serum- and anchorage-independent growth (Neufeld *et al*, 1988), demonstrating the oncogenic potential of FGF2.

Nearly 15 years ago, Schweigerer and co-workers isolated FGF2 from Ewing tumour cells. Unexpectedly, they demonstrated that this classical growth and differentiation factor induced growth arrest rather than proliferation in Ewing tumour cells *in vitro* (Schweigerer *et al*, 1987). Recent investigations showed that this growth arrest is accompanied by features characteristic of apoptotic cell death (Sturla *et al*, 2000; Westwood *et al*, 2002).

These results led to the question how Ewing tumour cells survive in an FGF2-containing environment *in vivo*? Modulation of FGF2-induced apoptosis by potential survival signals was the primary focus of this study. It has recently been pointed out that standard monolayer cultures may only poorly reflect the *in vivo* environment of Ewing tumour cells (Lawlor *et al*, 2002). Only a limited number of signalling cascades, required for tumour cell growth and survival *in vivo*, are triggered. Therefore, in this study potential survival signals, for example, integrin-mediated adhesion or soluble growth factors, that may counteract FGF2-induced cell death were studied under different *in vitro* conditions including anchorage-independent colony and adherent monolayer cultures.

## MATERIALS AND METHODS

### Cell culture and chemicals

The Ewing tumour cell lines CADO-ES1 and RD-ES were purchased from the DSMZ, Braunschweig, Germany. The TC-71 cell line was provided by Professor TJ Triche (Whang-Peng *et al*, 1984). RM-82, VH-64, and WE-68 were established by one of the authors (van Valen, 1999). All cell lines were maintained in RPMI 1640 medium (Biochrom, Berlin, Germany) containing 10% FCS, 2 mM L-glutamine, 100 U ml^−1^ penicillin and 100 *μ*g ml^−1^ streptomycin (all Lifetechnologies, Karlsruhe, Germany) at 37°C in a humidified 5% CO_2_ atmosphere. Cells were cultured in collagen-coated 25 cm^2^ tissue culture flasks (Greiner Labortechnik, Solingen, Germany). All cell lines were regularly tested for mycoplasma contamination. Lyophilised FGF2 (Sigma, Deisenhofen, Germany) was dissolved in IMDM (Lifetechnologies) at 5 *μ*g ml^−1^ (aliquoted and stored at −20°C until required). TNF-related apoptosis-inducing factor (TRAIL, PeproTech Inc., Rocky Hill, NJ, USA) was dissolved in PBS at 50 *μ*g ml^−1^. The phosphoinositide 3-OH kinase (PI3K) inhibitor LY294002 and the caspase inhibitors Ac-DEVD-CHO, z-IETD-FMK and z-VAD-FMK (all from Calbiochem, Bad Soden, Germany) were dissolved in DMSO at 50, 20, 10 and 20 mM, respectively.

### *In vitro* growth assays

Anchorage-independent growth was analysed by plating Ewing tumour cells as a single-cell suspension in semisolid medium containing 0.9% (w v^−1^) methylcellulose in IMDM, 15% FCS (v v^−1^) and 0.5% (w v^−1^) BSA. For serum-free assays, cells were plated in semisolid medium containing 0.9% methylcellulose in IMDM, 2.5% (w v^−1^) BSA and a supplement including 25 *μ*g ml^−1^ insulin, 50 *μ*g ml^−1^ transferrin, 6 *μ*g ml^−1^ cholesterol and 14 *μ*g ml^−1^ linoleic acid (all purchased from Sigma). For each assay, 1 ml of the cell suspension was plated in duplicate, triplicate or quadruplicate into sterile 35 mm Petri dishes (Nunc, Karlsruhe, Germany) that were incubated at 37°C. Colonies were counted after 11–14 days and each experiment was repeated at least two times.

Growth characteristics of adherent cells were investigated by utilising the assay described by Hansen *et al* (1989). 2 × 10^4^ cells were plated in quadruplicate in 100 *μ*l of RPMI 1640, 10% FCS, L-glutamin, penicillin and streptomycin or, for serum-free conditions, in 100 *μ*l IMDM containing 2% BSA (w v^−1^) and supplemental factors (as described above) in collagen-coated 96-well plates (Becton Dickinson, Heidelberg, Germany). Cells were allowed to attach for 3 h at 37°C with 5% CO_2_. Afterwards FGF2 was added. In experiments using caspase or PI3K inhibitors, these were added 1 h prior to FGF2 treatment. After different time intervals (24–72 h), 20 *μ*l of a 5 mg ml^−1^ MTT solution (3-(4,5-dimethylthiazol-2-yl)-2,5-diphenyltetrazolium bromide; Sigma) was added for 4 h at 37°C to each well. The media were replaced by 100 *μ*l of lysis solution (50% (v v^−1^) *N*,*N*-dimethylformamide, 20% (w v^−1^) SDS) (to dissolve the formazan crystals) and plates were read at 550 nm (test wavelength) and 630 nm (reference wavelength) in a Dynatech MR 7000 microplate reader (Denkendorf, Germany). Each experiment was repeated at least two times. Results are expressed as percentage of controls. The paired *t*-test was performed for statistical analysis.

### Detection of FGF2 protein

Crude cell lysates were obtained by resuspending 4 × 10^6^ cells in 1 ml extraction buffer (100 mM Tris–HCl, pH 8.0, 0.5% Triton X-100, 10 mM EDTA). Samples were subjected to three rounds of 5 min in liquid nitrogen, 5 min at 37°C and 5 min on ice. Samples were centrifuged, aliquoted and stored at −20°C. For analysis of FGF2 protein in crude cell lysates and supernatant, a FGF2-ELISA (R&D, Wiesbaden-Nordenstadt, Germany) was applied.

### Characterisation of cell death

Cells were grown in collagen-coated 25 cm^2^ tissue culture flasks containing media and 5 ng ml^−1^ FGF2 for up to 48 h. Cells were harvested and labelled using an annexin V-FITC apoptosis detection kit (Pharmingen, Heidelberg, Germany). Annexin V-positive cells were quantified by flow cytometry on a FACScan (Becton Dickinson) using CellQuest software (Becton Dickinson).

## RESULTS

### Fibroblast growth factor inhibits proliferation of clonogenic Ewing tumour cells

It has been well established that FGF2 inhibits proliferation of Ewing tumour cell lines *in vitro* (Schweigerer *et al*, 1987; Sturla *et al*, 2000; Westwood *et al*, 2002) though conflicting results have recently been reported under serum-free culture conditions (Girnita *et al*, 2000). To quantify this inhibitory effect of FGF2 on clonogenic tumour cells, the cell line TC-71 was plated in methylcellulose at different concentrations of FGF2.

**Figure 1:**
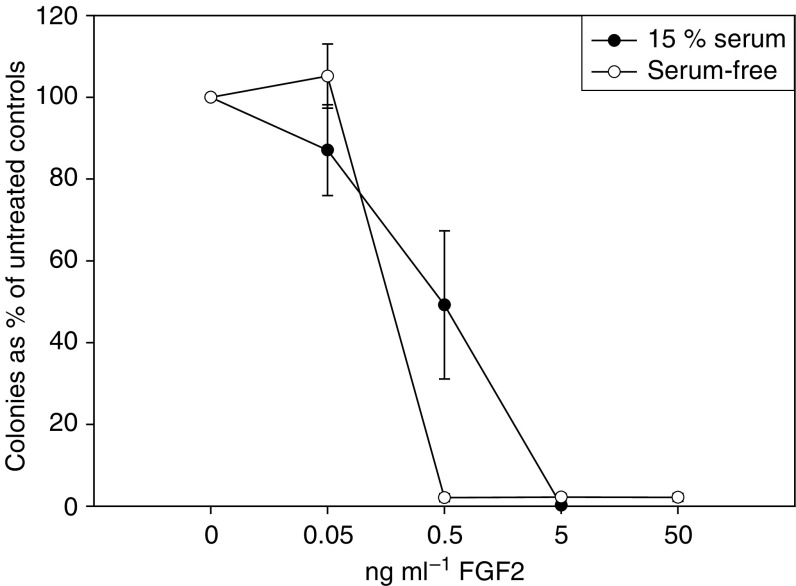
Increasing concentrations of FGF2 decreased the colony-forming potential of TC-71 cells in serum-containing (black dots) and serum-free (white dots) colony assays. This inhibitory effect was significant (*P*<0.05) for concentrations >0.5 ng ml^−1^. Notably, 15% FCS protected approx. 55% of the colony-forming ability of TC-71 at low concentrations of FGF2 (0.5 ng ml^−1^).

Under serum-free conditions, FGF2 at concentrations ranging from 50 down to 0.5 ng ml^−1^ (Figure 1) completely abrogated the colony-forming ability of TC-71 cells. However, in the presence of 15% FCS, TC-71 cells were less sensitive to 0.5 ng ml^−1^ FGF2. This protective effect of serum could be overcome by higher concentrations of FGF2. No stimulatory response to FGF2 at concentrations as high as 50 ng ml^−1^ was observed under any conditions.

### Ewing tumour cells express FGF2 as well as its receptors

Expression of FGF2 was assessed in a panel of Ewing tumour cell lines. Fibroblast growth factor protein could be detected in crude cell lysates from all the six cell lines, while no FGF2 protein could be detected in the supernatant of these cells (Table 1).

In order to identify the receptor(s) that transduces the inhibitory FGF2 signal in Ewing tumour cells, Western blot analyses identified FGFR1, -2 protein in all cell lines, whereas FGFR-3 protein was only detected in TC-71, VH-64 and WE-68. No FGFR-4 protein was found despite the presence of FGFR4-mRNA (data not shown). The Western blot results, therefore, confirm and extend previous studies (Sturla *et al*, 2000) by detection of FGFR1, -2 and -3 on the protein level in a high percentage of Ewing tumour cell lines.

### Fibroblast growth factor-induced growth arrest is accompanied by caspase-dependent apoptosis

In order to analyse the events accompanied by FGF2-induced growth inhibition, FGF2-treated cells were studied for markers of apoptosis. An early feature of apoptotic cells is exposure of phosphatidyl serines on the cell surface, which are recognised by the annexin V antibody. Consistent with published reports (Westwood *et al*, 2002), an annexin V single-positive population could be detected after 24 h treatment with FGF2 (data not shown).

**Figure 2:**
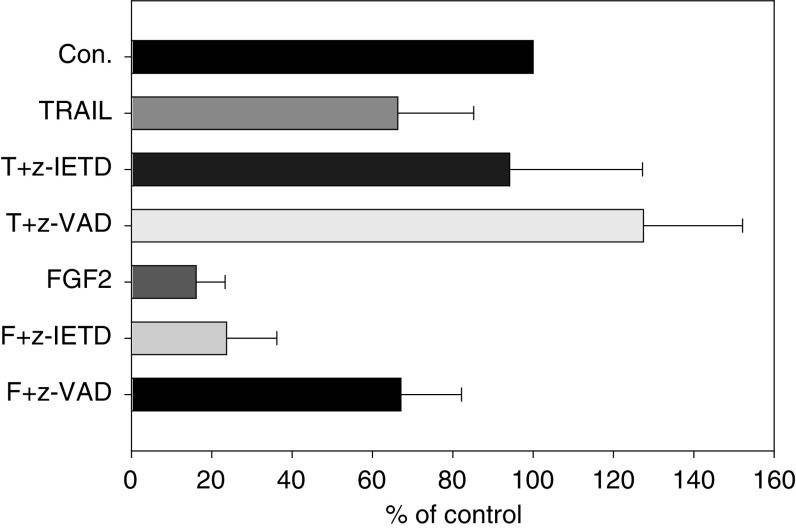
Both FGF2 and TRAIL inhibited proliferation of TC-71 Ewing tumour cells in serum-free monolayer cultures by induction of apoptosis. While TRAIL-induced apoptosis could be blocked both by the caspase 8 inhibitor z-IETD-FMK and pan-caspase inhibitor z-VAD-FMK, FGF2-induced apoptosis was only significantly inhibited by z-VAD-FMK (*P*<0.005). This indicates that the FGF2-induced death signal is not mediated via caspase 8. Con.: untreated control; TRAIL: 50 ng ml^−1^ TRAIL; T+z-IETD: 25 *μ*M z-IETD-FMK 1 h before TRAIL; T+z-VAD: 50 *μ*M of z-VAD-FMK 1 h before TRAIL; FGF2: 5 ng ml^−1^ FGF2; F+z-IETD: 25 *μ*M z-IETD-FMK 1 h before FGF2; F+z-VAD: 50 *μ*M of z-VAD-FMK 1 h before FGF2.

Caspases are essential proteases during apoptotic cell death. In order to define whether caspases are involved in FGF2-induced apoptosis, a panel of caspase inhibitors were applied: z-VAD-FMK – a pan-caspase inhibitor; Ac-DEVD-CHO – primarily an inhibitor of the effector caspase 3 and, to a lesser extent, caspase 7 and 8; z-IETD-FMK – an inhibitor of the initiator caspase 8. As shown in Figure 2, pre-incubation of TC-71 cells with these inhibitors did not completely protect TC-71 cells from FGF2-induced cell death: z-VAD rescues approx. 67% (67.1±15.1%) of the cells, the caspase 3 inhibitor Ac-DEVD-CHO protects only 35% (34.8±12.5%, data not shown) of the cells from apoptosis. In contrast, the caspase 8 inhibitor as well as the pan-caspase inhibitor were able to completely protect TC-71 cells from TRAIL-induced apotosis (Figure 2), indicating the effectiveness of the used inhibitors. These results confirm that FGF2-induced cell death is in part mediated via caspase activation. Interestingly and in contrast to TRAIL-induced apoptosis, the caspase 8 inhibitor z-IETD-FMK has no protective effect against FGF2-induced apoptosis (Figure 2).

### Adequate survival signals rescue Ewing tumour cells from FGF2-induced apoptosis

The obtained data confirm and extend recent findings showing that Ewing tumour cells express FGF2 and its receptors and that extracellularly supplied FGF2 induces apoptosis (Schweigerer *et al*, 1987; Sturla *et al*, 2000; Westwood *et al*, 2002). This leads to the central question how Ewing tumour cells survive and proliferate in an FGF2-containing environment *in vivo*.

To address this question, Ewing tumour cell survival in the presence of FGF2 was studied under different culture conditions. Monolayer cultures on collagen-coated culture flasks under defined serum-free conditions were used as a model for integrin-mediated survival signals. Spheroid cell cultures are regarded as suitable models for three-dimensional growth and cell–cell interactions of *in vivo* tumours (Santini and Rainaldi, 1999). Unlike the spheroid liquid-overlay cultures that allow aggregation of tumour cells (Santini and Rainaldi, 1999; Lawlor *et al*, 2002), the methylcellulose assay reads out single cells with the ability of clonal expansion, an important feature of metastatic cells.

**Figure 3:**
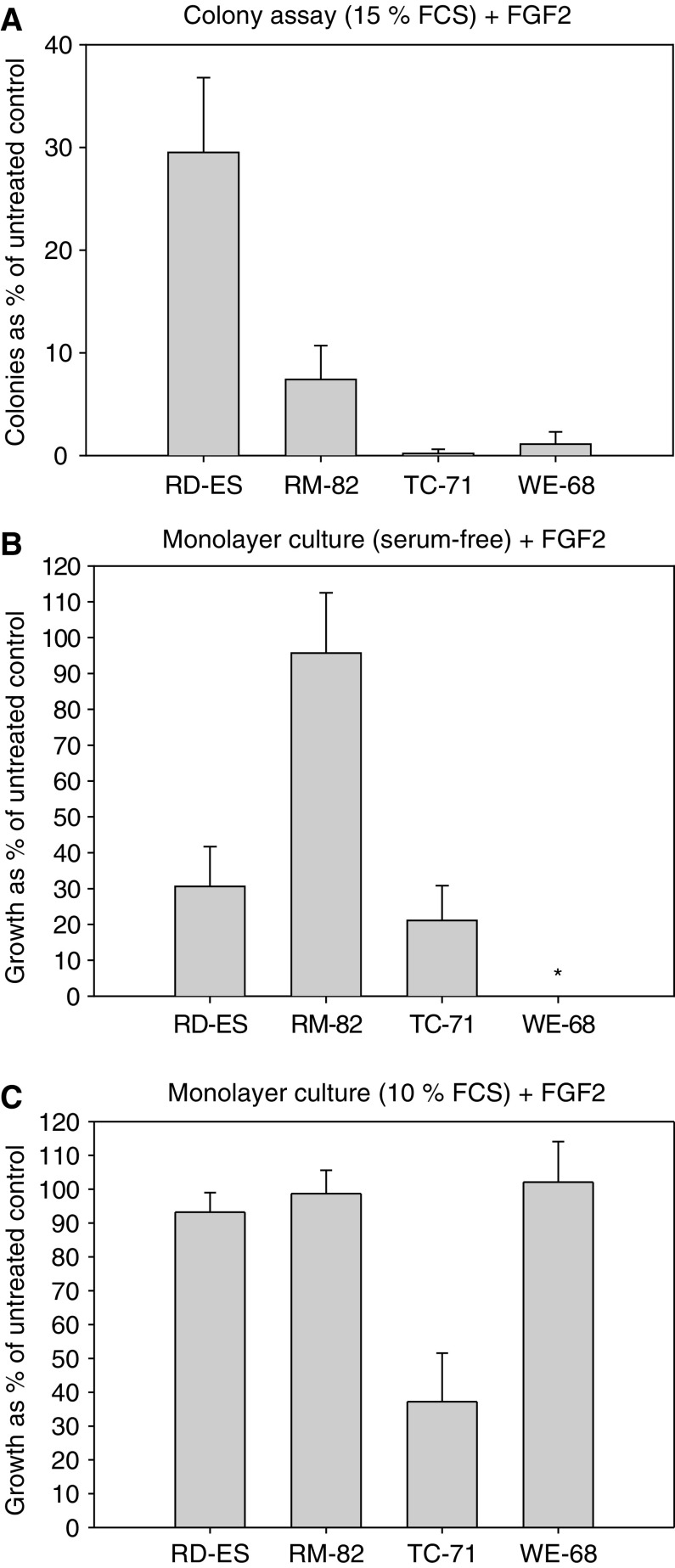
Comparison of the effects of 5 ng ml^−1^ FGF2 (**A**) in serum-containing (15% FCS) colony assays, (**B**) in serum-free adherent monolayer cultures and (**C**) in serum-containing adherent monolayer cultures. Integrin-mediated adhesion plus soluble serum factors had a strong synergistic effect in antagonising FGF2-induced apoptosis (**C**). ^*^WE-68 cells do not grow in serum-free adherent monolayer cultures.

Two cell lines (CADO-ES1 and VH-64) were resistant to FGF2 under all culture conditions (data not shown). Four cell lines (RD-ES, RM-82, TC-71 and WE-68) were FGF2-sensitive, but distinct culture conditions were able to rescue these cell lines from FGF-2-induced apoptosis. Exposure to serum alone (in the presence of 15% foetal calf serum) or integrin-mediated adhesion (serum-free monolayer cultures on a collagen matrix), both rendered one of the four Ewing tumour cell lines at least partially resistant to 5 ng ml^−1^ FGF-2 (serum in RD-ES and adhesion in RM-82; Figure 3A and B). However, integrin-mediated adhesion combined with serum (monolayer cultures on a collagen matrix with 15% FCS) provides a strong synergistic survival signal: under these conditions, three out of the four cell lines are completely resistant to 5 ng ml^−1^ FGF2 and 37.2±10.3% of TC-71 cells are protected from FGF2-induced apoptosis (Figure 3C).

Previous studies suggested insulin-like growth factor 1 (IGF1) as one of the key growth factors of Ewing tumours (van Valen *et al*, 1992; Scotlandi *et al*, 1996, 2002). It was, therefore, investigated whether serum-derived IGF1 may be the/one of the protective factor/s antagonising FGF2. However, IGF1 at 0.2–20 ng ml^−1^ neither increased the colony-forming ability and proliferative activity of Ewing tumour cells under serum-free conditions, nor was IGF1 sufficient to protect TC-71 cells from FGF2-induced apoptosis (data not shown).

### Phosphoinositide 3-OH kinase is involved in integrin- and serum-mediated survival signals

The PI3K/AKT pathway is considered to be an important signalling pathway mediating survival signals in Ewing tumour cells. Activation of AKT leads to phosphorylation and inactivation of proapoptotic molecules such as bad, forkhead and caspase 9, and activation of molecules regulating cell growth and expression of genes responsible for survival (Datta *et al*, 1999). Inhibition of this signalling pathway in Ewing tumour cells with the PI3K specific inhibitor LY294002 has been reported to enhance apoptosis (Toretsky *et al*, 1999). In addition, it has been shown that integrin signalling renders tumour cells insensitive to apoptosis-inducing drugs (Aoudjit and Vuori, 2001) and this resistance is also mediated by the PI3K/AKT pathway.

**Figure 4:**
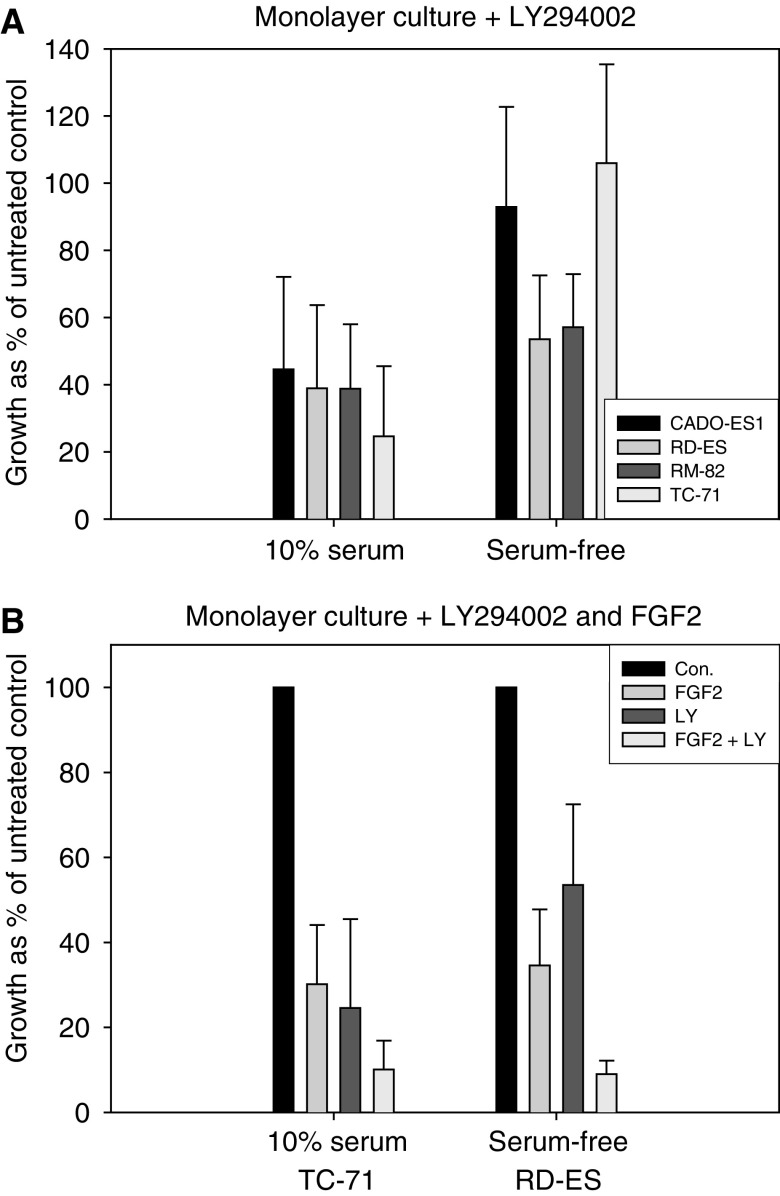
Inhibition of PI3K with LY294002 (80 *μ*M) decreased proliferation/growth of all Ewing tumour cell lines to approx. 38% in serum-containing adherent cell cultures (*P*<0.05) (**A**) (left columns). Under serum-free conditions, no significant inhibitory effect was seen with the cell lines CADO-ES1 and TC-71 (*P*>0.7), while RD-ES and RM-82 showed a reduction in proliferation to approx. 45% (**A**) (right columns). The combination of LY294002 and FGF2 resulted in an increased sensitivity to FGF2 in serum-containing (TC 71 cells) (**B**) (left columns) and in serum-free adherent monolayer cultures (RD-ES cells) (**B**) (right columns) (*P*<0.05). This indicates that PI3K-mediated survival signals are involved in antagonising FGF2-induced apoptosis.

Therefore, it was expected that this pathway may also be involved in serum- and adhesion-mediated resistance to FGF2-induced apoptosis. LY294002 is a specific PI3K inhibitor (Vlahos *et al*, 1994) and its interaction with the ATP-binding pocket of PI3K has been well characterised (Walker *et al*, 2000). When 80 *μ*M LY294002 was added to serum-containing adherent monolayer cultures, tumour cell proliferation was dramatically reduced to approximately 40% in all four cell lines tested (Figure 4A). Under serum-free adherent monolayer culture condition, LY294002 had no effect on tumour cell proliferation in CADO-ES1 and TC-71 cells, but a minor inhibitory effect on RD-ES and RM-82 cells (Figure 4A). This indicates that PI3K signalling is central in mediating serum-related survival signals in all cell lines as well as in mediating adhesion-related survival signals in RD-ES and RM-82 cells.

In accordance with this assumption are the results obtained when TC-71 and RD-ES cells were cultured in the presence of FGF2 and LY294002 under serum-containing and serum-free conditions (Figure 4B). This combination resulted in an increased effect on FGF2-induced apoptosis under serum-containing culture conditions in TC-71 cells (Figure 4B left columns), indicating that inhibition of the serum-triggered PI3K survival signal renders TC-71 cells more sensitive to FGF2-induced apoptosis. A similar effect was seen with RD-ES cells. When these cells were cultured under serum-free conditions, the inhibition of the adhesion-triggered PI3K survival signal resulted in sensitisation of these cells to FGF2-induced apoptosis (Figure 4B, right columns).

These results show that PI3K signalling plays an important role in mediating serum- and/or integrin-related survival signals in Ewing tumour cell lines and that these survival pathways are involved in antagonising FGF2-induced apoptosis.

## DISCUSSION

We were able to confirm that FGF2 has a dose-dependent growth-inhibitory effect on most Ewing tumour cell lines *in vitro* and that this growth inhibition is associated with induction of apoptosis. However, the biological and clinical impact of expression of FGF2 and its receptors by Ewing tumour cells remains obscure.

Activation of initiator caspases in FGF2-treated cells, including the receptor-triggered caspase 8 and lack of mitochondrial cytochrome *c* release, had led to the hypothesis that FGF2-induced apoptosis may represent a new classical receptor-induced cell death pathway (Westwood *et al*, 2002). If this hypothesis is true, a caspase 8 inhibitor would be expected to protect Ewing tumour cells from FGF2-induced apoptosis. However, this assumption was not supported by our experiments. Inhibition of the receptor-associated initiator caspase 8 with z-IETD-FMK had no effect on Ewing tumour cell survival after exposure to FGF2. The delay in the occurrence of cell death after exposure to FGF2 indicates that induction of apoptosis may be a secondary event mediated by an unknown signalling cascade. Recently, it has been found out that FGF2-induced activation of JNK may play a crucial role in inducing apoptosis in Ewing sarcoma-related Askin tumour cells (Kim *et al*, 2004).

Nevertheless, four out of six Ewing tumour cell lines *in vitro* die after exposure to FGF2. In this context, it was surprising to observe that all Ewing tumour cell lines expressed FGF2 at the RNA and protein level (data not shown and Table 1). In contrast to L87/4 stroma cells, however, no FGF2 could be detected in the culture medium of the Ewing tumour cell lines (Table 1). Fibroblast growth factor lacks a secretory signal and is secreted by an ER/Golgi-independent mechanism (Engling *et al*, 2002). The lack of secretion of FGF2 under standard culture conditions may be one of the mechanisms by which Ewing tumour cells protect themselves from autocrine growth inhibition and induction of apopotosis.

However, lack of secretion of FGF2 by Ewing tumour cells is insufficient to explain formation, survival and metastasis of these tumour cells in or to the bone marrow microenvironment, which is a rich source of FGF2 (Brunner *et al*, 1993). Similar to the situation in patients, Ewing tumours metastasise at high frequency to bone and bone marrow in NOD/*scid* mice (Vormoor *et al*, 2001), indicating that the bone marrow microenvironment is supportive for growth and survival of these cells. It was, therefore, hypothesised that this FGF2-containing microenvironment itself is responsible for rendering Ewing tumour cells insensitive to FGF2. The complex interaction between tumour cells and their microenvironment and the importance of this interaction for tumour cell survival is increasingly been recognised (Liotta and Kohn, 2001). In this context, it has been pointed out that standard monolayer cultures may only poorly reflect the *in vivo* environment of Ewing tumour cells (Lawlor *et al*, 2002), emphasising the importance of culture conditions on *in vitro* results and their biological significance.

Using both adherent monolayer and non-adherent colony assays allowed dissection of important aspects of the interaction between Ewing tumour cells and their microenvironment. Both integrin-mediated adhesion and soluble serum factors alone provide positive, though weak, survival signals partially antagonising FGF2 activity. However, adhesion combined with serum had a strong synergistic effect and caused Ewing tumour cell resistance to FGF2-induced cell death. These latter conditions most likely best reflect the microenvironment of human Ewing tumours *in vivo*, suggesting that FGF2-induced apoptosis may be a tissue culture artefact.

The serum as well as the adhesion signal is mediated to a large extent by the classical PI3K signalling cascade, as could be shown by inhibition experiments with LY294002. Phosphoinositide 3-OH kinase is a key regulator of growth factor- and integrin-related survival signals and the role of its downstream target, the kinase AKT, has been well established in preventing apoptosis and supporting survival of many cell types (Datta *et al*, 1999; Danen and Yamada, 2001). However, it remains to be identified which soluble growth factors are responsible for this serum effect. The serum effect could not be attributed to IGF1, a growth factor thought to be one of the key mitogenic and survival factors in Ewing tumours. In addition, the resistance of Ewing tumour cells to imatinib mesylate, an inhibitor of c-KIT (Hotfilder *et al*, 2002), suggests that stem cell factor is also not a key survival factor in Ewing tumours.

Although it could be demonstrated that Ewing tumour cells escape FGF2-induced apoptosis through microenvironment-mediated survival signals, the biological function of FGF2 expression in Ewing tumour cells remains unknown. Under serum-free conditions, FGF2 has been shown to be a potent inducer of EWS/FLI1 protein expression and by this mechanism may stabilise the malignant phenotype (Girnita *et al*, 2000). Alternatively, FGF2 may be released under hypoxic stress as a potent angiogenic factor; it may affect bone remodelling and thus tumour progression by osteoclast recruitment (Collin-Osdoby *et al*, 2002), or it may act in a paracrine fashion by inducing growth factor production in bone marrow stroma cells (Zhang *et al*, 2002; Bisping *et al*, 2003).

In conclusion, although the biological function of FGF2 expression remains to be defined, several mechanisms could be identified by which Ewing tumour cells escape the detrimental effects of FGF2. These include the absence of FGF2 secretion under standard conditions and the modulation of inhibitory FGF2 signalling pathways by survival signals provided from integrin-dependent adhesion and soluble serum factors. The PI3K/AKT pathway is a key pathway involved in mediating the survival signals.

These survival signals need further investigation as they can be expected to play a central role in resistance to other apoptosis-inducing agents including chemotherapeutic drugs (Aoudjit and Vuori, 2001) and will help to understand how Ewing tumour cells survive under sometimes hostile conditions *in vivo*. A better understanding of these survival signals will facilitate the development of treatments more specifically targeted against the tumour cells.

## Figures and Tables

**Table 1:** Detection of FGF2 in cell lysates and supernatants in a panel of Ewing tumour cell lines

	**Protein in**	
**Cell line**	**Supernatant pg/10^6^ cells**	**Cell lysate pg/10^6^ cell**
CADO-ES1	—a	47.3±6.9
RD-ES	—	0.75±0.009
RM-82	—	20.7±1.1
TC-71	—	35.5±4.1
VH-64	—	9.2±0.4
WE-68	—	78.9±0.6
L87/4 (BM stroma)	278.3±21.7	3422±110
Medium (10% FCS)	—	
Medium (0% FCS)	—	

a –: values obtained were below the detection limit of 10 ng ml^−1^.
